# Editorial: Exploring the genetic potential of *Thinopyrum* species in wheat and durum wheat improvement

**DOI:** 10.3389/fpls.2023.1332662

**Published:** 2023-11-23

**Authors:** Edina Türkösi, Klaudia Kruppa, Mahmoud Said

**Affiliations:** ^1^Department of Biological Resources, Centre for Agricultural Research, Martonvásár, Hungarian Research Network (HUN-REN), Martonvásár, Hungary; ^2^Institute of Experimental Botany of the Czech Academy of Sciences, Centre of Plant Structural and Functional Genomics, Olomouc, Czechia; ^3^Field Crops Research Institute, Agricultural Research Centre, Giza, Cairo, Egypt

**Keywords:** wheat, *thinopyrum*, introgression breeding, *in situ* hybridization (ISH) analysis, marker assisted selection, pollen dispersal, seed trait variability

Wheat is one of the most significant grain crops, with around 220 million hectares under cultivation worldwide. It plays an important role for ensuring food security. However, wheat domestication and breeding activities have created a genetic bottleneck, drastically limiting its future progress. Wheat wild relatives, on the other hand, were not subjected to human selection and constitute a vast reservoir of agronomically beneficial genes that can be introgressed into wheat using chromosome engineering techniques. Wheatgrass (*Thinopyrum*) species are related to cultivated wheat and exhibit considerable genetic variability suitable for wheat breeding.

Due to the availability of genetic stocks bearing its chromatin, *Thinopyrum intermedium* (intermediate wheatgrass) is one of the most often used wheatgrass species for chromosome engineering and commonly used to introgress disease-resistance genes into wheat. Transferring foreign chromosome segments into wheat may cause linkage drag. Thus, partial amphiploids, additions, and substitution lines are only used as bridge material in introgression breeding with the goal of reducing the amount of alien DNA and establishing introgression lines. Two translocation lines were produced by irradiating the pollen of the stripe rust-resistant wheat-*Th. intermedium* addition line TAI-14 (Guo et al.). The segmental translocation line Zhongke 78 and the intercalary translocation line Zhongke 15 both demonstrated high resistance to stripe rust at the seedling and adult plant stages. Furthermore, Zhongke 15 is an insertion of wheatgrass chromatin into the satellite of chromosome 6B, a type of translocation that has never been described before. Fourteen markers specific for the introgressed chromosome segment were identified by screening Simple Sequence Repeat (SSR), Expressed Sequence Tag (EST), and markers developed from RNA-sequencing (RNA-Seq) data, as well as the marker T14K50 linked to the *YrT14* resistance gene, which can be used for marker-assisted selection of resistant genotypes. Based on the karyotype, reaction to fungus, and genome resequencing data the stripe rust resistance gene *YrT14* was found to be located on an 88.1 Mb interval ranging from 636.7 to 724.8 Mb on *Th. intermedium* chromosome corresponding to 7J or 7J^s^.

*Th. intermedium* was the first wheatgrass to be domesticated as a grain crop due to its low seed shattering, low lodging, and good threshability when compared to other perennial grasses, as well as producing reasonably large edible seed with synchronous maturation (Wagoner, 1990). Given that genetic diversity of plant populations is a decisive feature in crop breeding programs, Bajgain et al. conducted research in an advanced breeding germplasm to study pollen dispersal and assess its effect on genetic diversity and trait distribution in the progeny. Using genome-wide SNP markers to estimate population diversity, investigating pollen dispersal patterns, and assigning paternity in the breeding population, it was determined an average heterozygosity of 0.39, which was higher than that of other perennial grasses. Mating success was the highest among close-range genets and decreased as distance increased. There was no significant correlation found between the father genets’ morphological traits and their pollination success. Similarly, there was a low correlation between parental traits and pollination distance. The outcrossing rate was outstanding, with inbreeding almost non-existent (<1%). The results of the research group indicated that the distance of fathers from the mother plants had no effect on the diversity and trait distribution of progeny for essential domestication features. At the same time, the findings validate the research group’s current method of progeny selection using predicted *per se* performance, that is, best values for traits of interest predicted by genomic selection models can be continued without significantly reducing future population genetic diversity.

Soil salinization has recently emerged as a serious environmental concern worldwide, and it is likely to worsen further (Hassani et al., 2021). Wheatgrass species with E genomes (for example, halophile wheatgrass *Th. elongatum*) and *Tritipyrum*, derived from wide crosses of *Triticum* and *Th. elongatum*, have salt-tolerant properties, making them rich sources of genetic variability for wheat improvement (Margiotta et al., 2020). Late embryogenesis-abundant (LEA) proteins play an important role in plant abiotic stress tolerance, particularly tolerance to soil salinity. Yang et al. described the cloning and characterization of a novel LEA gene (*TtLEA2-1*) from *Tritipyrum* “Y1805”, including its sequence, evolutionary relationships, expression patterns, and interacting proteins under salt stress. The phylogenetic study revealed that *TtLEA2-1* is derived from the E genome of *Th. elongatum*. Following the screening of 48 potential proteins hypothesized to interact with *TtLEA2-1*, it was assessed that these proteins were primarily enriched in environmental information processing, glycan biosynthesis and metabolism, and carbohydrate metabolism pathways. Researchers developed a robust and reproducible wheat transformation system (coleoptile meristem infiltration) and demonstrated that transgenic wheat variety carrying the *TtLEA2-1*gene grew taller, had stronger roots and higher catalase activity than wild-type plants under 250 mM NaCl stress, demonstrating efficient transformation and the role of the novel gene in salt tolerance.

Tall wheatgrass (*Th. ponticum*), which has been shown to be highly resistant to powdery mildew (Zhang et al., 1996; Chen et al., 1998; Li and Wang, 2009), has been widely used in wheat genetic improvement. Yang et al. developed four wheat-*Th. ponticum* translocation lines carrying varied segments from tall wheatgrass chromosome 4Ag by using irradiated pollen from the Blue 58 [4Ag (4D) disomic substitution] line. Powdery mildew experiments conducted over two growing seasons revealed that at the adult plant stage, three translocation lines (WTT139, WTT146, and WTT323) were highly resistant to *Blumeria graminis* f. sp. *tritici* (Bgt) race E09. The *Pm* locus giving adult plant resistance was mapped to the 3.79-97.12 Mb region on the short arm of chromosome 4Ag by integrating the results of resistance evaluation, particular marker amplification, and alignment analysis. The powdery mildew resistance locus was introgressed into wheat cultivar Jimai 22 to improve the translocation lines’ poor agronomic traits. There is no evidence of linkage drag associated with the alien segment, making this line potentially effective for enhancing powdery mildew resistance in wheat.

An international research team investigated seed trait variability and its environmental dependence across a latitudinal gradient in two main engineered species (*Th. junceum* and *Calamagrostis arenaria*) of European dune systems found along the coasts of the Mediterranean Basin, Atlantic Ocean, and North Sea Del Vecchio et al.. The seed germination responses to temperature and seed mass variation within and across the populations exposed to a gradient of temperature and precipitation regimes were examined at six different locations. It was determined that seed germination responses to temperature of *Th. junceum* and *C. arenaria* from different populations differed significantly based on seed origin. Nonetheless, seed germination responses were only partially correlated with the populations’ latitudinal climate gradient, using diverse germination techniques. It was determined that seed germination was warm-cued in populations exposed to harsh winters, whereas seed germination was cold-cued in populations from warm locations with dry summers. The scientists’ research on seed mass variability at the within-species level suggested that the effect of climatic variables on this trait is not clearly predictable, because seed trait variation may show different responses than those expected by analyzing the climatic features of the seed site of origin. The researchers’ findings could help uncovering previously unknown patterns of species dynamics, allowing suitable actions to offset biodiversity loss to be planned.

Wheatgrass species have been the focus of breeders’ attention for decades, with the goal of harnessing their genetic potential to broaden wheat diversity. Implementation of marker assisted selection allows efficient screening of agronomically useful traits introgressed from donor germplasm into elite breeding lines. Furthermore, population genetics research helps to improve understanding of genetic diversity in breeding populations and seed characteristic variations throughout the latitudinal gradient. Increased availability of wild relatives’ accessions in gene banks, improved interspecific hybridization procedures, and advanced molecular technologies may lead to an expansion of the scale of agronomic features introgressed from wheatgrass species into cultivated wheat.

## Author contributions

ET: Writing – original draft, Writing – review & editing. KK: Writing – original draft, Writing – review & editing. MS: Writing – original draft, Writing – review & editing.
